# Infections in pediatric acute promyelocytic leukemia: from the canadian infections in acute myeloid leukemia research group

**DOI:** 10.1186/1471-2407-13-276

**Published:** 2013-06-04

**Authors:** Sonia Cellot, Donna Johnston, David Dix, Marie-Chantal Ethier, Biljana Gillmeister, David Mitchell, Rochelle Yanofsky, Victor Lewis, Carol Portwine, Victoria Price, Shayna Zelcer, Mariana Silva, Lynette Bowes, Bruno Michon, Kent Stobart, Josee Brossard, Joseph Beyene, Lillian Sung

**Affiliations:** 1 Hematology/Oncology, Hospital Sainte-Justine, 3475 Chemin Cote Ste-Catherine, Montreal, QC, H3T 1C5, Canada; 2 Hematology Oncology, Children’s Hospital of Eastern Ontario, 401 Smyth Road, Ottawa, ON, K1H 8L1, Canada; 3 Pediatric Hematology/Oncology, British Columbia Children’s Hospital, 4480 Oak Street, Vancouver, BC, V6H 3N1, Canada; 4 Child Health Evaluative Sciences, The Hospital for Sick Children, 555 University Avenue, Toronto, ON, M5G 1X8, Canada; 5 Hematology/Oncology, Montreal Children’s Hospital, 2300 rue Tupper, Montreal, QC, H3H 1P3, Canada; 6 Hematology/Oncology, CancerCare Manitoba, 675 McDermot Avenue, Winnipeg, MB, R3E 0V9, Canada; 7 Hematology/Oncology/Transplant Program, Alberta Children’s Hospital, 2888 Shaganappi Trail NW, Calgary, AB, T3B 6A8, Canada; 8 Hematology/Oncology, McMaster Children’s Hospital at Hamilton Health Sciences, 1280 Main Street West, Hamilton, ON, L8S 4K1, Canada; 9 Pediatrics, IWK Health Centre, 5850/5980 University Avenue, P.O. Box 9700, Halifax, NS, B3K 6R8, Canada; 10 Hematology/Oncology, London Health Sciences, 800 Commissioner’s Road East, London, ON, N6C 2V5, Canada; 11 Hematology/Oncology, Cancer Centre of Southeastern Ontario at Kingston, 25 King St, W Kingston, ON, K7L 5P9, Canada; 12 Hematology/Oncology, Janeway Child Health Center, 300 Prince Philip Drive, St. John’s, NF, A1B 3V6, Canada; 13 Pediatric Hematology/Oncology Centre, Hospitalier Universitaire de Quebec, 2705 Boulevard Laurier, Quebec, QC, G1V 4G2, Canada; 14 Stollery Children’s Hospital, University of Alberta Hospital, 11405 87 Ave, Edmonton, AB, T6G 1C9, Canada; 15 Hematology/Oncology, Centre Hospitalier Universitaire de Sherbrooke, 3001 12e Avenue Nord, Sherbrooke, QC, J1H 5N4, Canada; 16 Population Genomics Program, Department of Clinical Epidemiology and Biostatistics, McMaster University, 1280 Main Street West, Hamilton, ON, L8S 4K, Canada; 17 Division of Haematology/Oncology, The Hospital for Sick Children, 555 University Avenue, Toronto, ON, M5G 1X8, Canada

**Keywords:** Infection, Acute promyelocytic leukemia, Bacteremia, Sepsis, Acute myeloid leukemia

## Abstract

**Background:**

It is not known whether children with acute promyelocytic leukemia (APL) have an infection risk similar to non- APL acute myeloid leukemia. The objective was to describe infectious risk in children with newly diagnosed APL and to describe factors associated with these infections.

**Methods:**

We conducted a retrospective, population-based cohort study that included children ≤ 18 years of age with *de novo* APL treated at 15 Canadian centers. Thirty-three children with APL were included; 78.8% were treated with APL -specific protocols.

**Results:**

Bacterial sterile site infection occurred in 12 (36.4%) and fungal sterile site infection occurred in 2 (6.1%) children. Of the 127 chemotherapy courses, 101 (79.5%) were classified as intensive and among these, the proportion in which a sterile site microbiologically documented infection occurred was 14/101 (13.9%). There was one infection-related death.

**Conclusions:**

One third of children with APL experienced at least one sterile site bacterial infection throughout treatment and 14% of intensive chemotherapy courses were associated with a microbiologically documented sterile site infection. Infection rates in pediatric APL may be lower compared to non- APL acute myeloid leukemia although these children may still benefit from aggressive supportive care during intensive chemotherapy.

## Background

Children with acute myeloid leukemia (AML) are at substantial risk of morbidity and mortality from invasive bacterial and fungal infections [1]. Even with this large infectious burden, there is great variability in supportive care strategies used for pediatric AML across institutions [2]. Clinical trials are currently being conducted to address these uncertainties [3,4]. However, there are some common themes; most North American centers do not use routine anti-bacterial prophylaxis (other than for *Pneumocystis jirovecii*) and most use fluconazole as antifungal prophylaxis [2].

Acute promyelocytic leukemia (APL) is a rare sub-type of AML, comprising only 5-10% of pediatric AML [5]. These children can have a specific and life-threatening clinical presentation consisting of hemorrhage and thrombosis, resulting in a relatively high induction death rate [6]. They also uniquely experience differentiating syndrome following exposure to all-trans-retinoic acid. Children with APL are typically excluded from AML supportive care clinical trials. It is unknown whether supportive care designed for non-APL AML patients should be applied to children with APL. We chose to describe and evaluate infectious toxicities in children with APL so as to understand whether they are similar to those in children with other pediatric AML.

Because of the rarity of pediatric APL and because these children are often very ill at presentation, there are much less data available on infections in APL derived from therapeutic clinical trials compared with non-APL AML clinical trials. Consequently, we conducted a population-based retrospective study in order to describe the risk of infection in these children. The primary objective was to describe infectious risk in children with newly diagnosed APL and to determine factors associated with infections in this population.

## Methods

This was a retrospective, population-based cohort study that included children with newly diagnosed APL treated at all 15 Canadian centers that care for children with cancer in each province except for Saskatchewan. This manuscript is related to a larger study in which children with newly diagnosed non-APL AML in Canada were examined [7].

### Study sample

We included children and adolescents diagnosed with *de novo* APL between January 1, 1995 and December 31, 2004 who were age ≤ 18 years at diagnosis and who received any treatment for APL. We excluded those with secondary APL and previous diagnosis of immunodeficiency.

### Outcome measures

Infections were examined from initiation of APL treatment until hematopoietic recovery from the last cycle of chemotherapy, conditioning for hematopoietic stem cell transplantation, relapse, persistent disease leading to a change in protocol therapy, or death (whichever occurred first). We used consistent trained clinical research associates to abstract and code the relevant information.

The rates of sterile site microbiologically documented infection [8], clinically documented infection and fever of unknown origin were expressed as the number of events during the time period at risk. Positive cultures with common contaminants such as coagulase negative *Staphylococcus* were only considered true infection if there were two or more positive cultures within the same episode or if the infection was associated with sepsis [9,10]. A patient was considered to have sepsis if there was systemic inflammatory response syndrome in the presence of suspected or proven infection and organ dysfunction according to international consensus guidelines [11,12]. Classification of clinically documented infections was based upon the Centers for Disease Control and Prevention definitions of nosocomial infections [13]. Fever of unknown origin was defined as a fever occurring in the absence of a positive microbiology result or clinical site of infection. Infections were evaluated separately among intensive and non-intensive courses. Induction and consolidation chemotherapy were considered intensive. Maintenance chemotherapy was considered non-intensive and the entire maintenance period was considered one treatment course as we were most interested in intensive treatment in terms of infection outcomes.

### Potential predictors

We chose to evaluate factors potentially associated with infection outcomes only among intensive chemotherapy courses as these are the most clinically relevant from an infection supportive care perspective. The following variables were evaluated: (1) Child characteristics at diagnosis (age and obese versus non-obese); (2) Treatment characteristics (APL-specific treatment protocol, registration on APL trial, diagnosis prior to January 1, 2000, and cumulative dose of cytarabine in grams/m^2^); (3) Course characteristics (neutropenia at the start of the course, neutropenia >15 days (threshold chosen *a priori*), and days systemic corticosteroids were administered for any reason).

Obesity was defined as a body mass index ≥ 95 percentile for age and gender according to the Centers for Disease Control and Prevention for those at least 2 years of age [14].

### Statistics

Regression analyses were conducted at the course level and only included intensive chemotherapy. Factors associated with rates of microbiologically documented sterile site infection, clinically documented infection and fever of unknown origin were examined using repeated measures Poisson regression and the association was expressed as a rate ratio (RR) with 95% confidence interval (CI). Multiple regression was conducted using variables significant in univariate analysis. In order to evaluate co-linearity and which variables should not be concurrently included in the multiple regression model, Spearman correlation coefficients (r) were examined. All tests of significance were two-sided, and statistical significance was defined as P <0.05. Statistical analysis was performed using the SAS statistical program (SAS-PC, version 9.3; SAS Institute Inc., Cary, NC).

### Ethical approvals

This study was approved by the Research Ethics Board at The Hospital for Sick Children and local Research Ethics Boards of the 14 other participating sites (McMaster University-Hamilton Health Sciences/Faculty of Health Sciences Research Ethics Board, Montreal Children’s Hospital Research Ethics Board, Children’s Hospital of Eastern Ontario Research Ethics Board, University of Winnipeg Research Ethics Board, University of British Columbia/Children’s and Women’s Health Centre of British Columbia Research Ethics Board, Centre Hospitalier Universitaire Sainte-Justine Research Ethics Board, University of Calgary Conjoint Health Research Ethics Board, IWK Research Ethics Board, Queen’s University-Health Sciences Research Ethics Board, University of Western Ontario Research Ethics Board for Health Science Research Involving Human Subjects, Memorial University Human Investigation Committee, Centre Hospitalier Universitaire de Quebec Research Ethics Board, University of Alberta Health Research Ethics Board-Biomedical Panel, Centre Hospitalier Universitaire de Sherbrooke Research Ethics Board). As this was a retrospective review study the Research Ethics Board at The Hospital for Sick Children and those at the 14 other participating sites waived the need for written informed consent.

## Results

In terms of demographics, of the 33 children with APL included in this analysis, most (78.8%) were treated according to APL-specific protocols (Table 1). The median days receiving intensive chemotherapy (from first to last administration) was 79 days (interquartile range (IQR) 67 to 104 days) and the median days receiving maintenance chemotherapy was 396 days (IQR 135 to 565 days). Among the 33 children, throughout the course of therapy, 12 (36.4%) experienced any bacterial sterile site infection and 2 (6.1%) experienced any fungal sterile site infection (both candidemia).

**Table 1:** Demographic and treatment characteristics for children with acute promyelocytic leukemia (N = 33)

	**Value**
**Child characteristics at diagnosis**	
Male (%)	16 (48.5)
Median age in years (IQR)	12.5 (7.2, 15.6)
Down syndrome (%)	1 (3.0)
Body mass index (%), N = 32^1^	
Obese	10 (31.2)
Normal weight	22 (68.8)
Median white blood cell count at diagnosis (×10^9^/L) (IQR)	4.8 (2.5, 15.0)
Median absolute neutrophil count at diagnosis (×10^9^/L) (IQR)^2^	0.2 (0.1, 1.1)
Cytogenetics (%)	
t(15;17) (q22;q12) (PML/RARα) and variants	28 (84.8)
Unknown	5 (15.2)
**Treatment characteristics**	
Protocol (%)	
APL-specific^3^	26 (78.8)
Non-APL specific AML protocol	7 (21.2)
Registered on study (%)	4 (12.1)

Abbreviations: *IQR* interquartile range, *APL* acute promyelocytic leukemia, *AML* acute myeloid leukemia; ^1^ One patient < 2 years of age at diagnosis; ^2^ ANC unavailable for two patients at diagnosis; ^3^ Includes APL standard of care (n = 1), Children’s Cancer Group 2911 (n = 5), Children’s Oncology Group A9710 (n = 20).

Table 2 illustrates the course characteristics and supportive care received among all 127 courses; 101 were classified as intensive (79.5%) and 99 were classified as APL specific (78.0%). Among intensive treatment courses, the proportion in which a sterile site microbiologically documented infection occurred was 14/101 (13.9%) while the number in which a sterile site fungal infection occurred was 2/101 (2.0%). The table also illustrates that there were 8 courses complicated by sepsis and 1 infection-related death due to *Klebsiella pneumoniae* bacteremia.

**Table 2:** Course characteristics and infection outcomes (N = 127)

	**All (N = 127)**	**Intensive treatment (N = 101)**	**APL-specific treatment (N = 99)**
**Course characteristics**			
Number with neutropenia (ANC <0.5 × 10^9^) at start of course (%)	27 (21.3)	27 (26.7)	21 (21.2)
Median days with neutropenia^1^(IQR)	7.5 (0.0, 19.0)	11.0 (0.0, 20.0)	3.0 (0.0, 18.0)
Median days receiving systemic corticosteroids (IQR)	0.0 (0.0, 5.0)	0.0 (0.0, 6.0)	0.0 (0.0, 3.0)
Median corticosteroid dose^1^ (IQR)	0.0 (0.0, 29.7)	0.0 (0.0, 38.4)	0.0 (0.0, 5.7)
**Supportive care**			
Co-trimoxazole prophylaxis (%)	89 (70.1)	71 (70.3)	77 (77.8)
Other antibacterial prophylaxis (%)	0	0	0
Fluconazole prophylaxis (%)	30 (23.6)	27 (26.7)	18 (18.2)
**Infection outcomes**^2^			
Sterile site microbiologically documented infection (%)	15 (11.8)	14 (13.9)	9 (9.1)
Sterile site Gram-positive (%)	12 (9.5)	11 (10.9)	7 (7.1)
Sterile site Gram-negative (%)	4 (3.2)	4 (4.0)	2 (2.0)
Sterile site fungus (%)	2 (1.6)	2 (2.0)	2 (2.0)
Bacteremia (%)	11 (8.7)	10 (9.9)	7 (7.1)
Clinically documented infection (%)	30 (23.6)	24 (23.8)	26 (26.3)
Sepsis	8 (6.3)	7 (6.9)	8 (8.1)
Infectious death	1 (0.8)	1 (1.0)	1 (1.0)

Abbreviations: *ANC* absolute neutrophil count, *IQR* interquartile range; ^1^ Presented as mg/m^2^ of dexamethasone equivalents; ^2^Infection outcomes represent at least one event per course; number of specific infections do not add to the total as 3 courses had multiple infection types.

Treatment with an APL-specific protocol was not associated with significantly fewer microbiologically documented sterile site infections when only intensive treatment courses were included (Table 3). Only treatment in an earlier era, and a higher cytarabine dose were associated with higher rates of infection. Neutropenia at the start of the course and prolonged neutropenia were both significantly associated with clinically documented infection (Table 3). Finally, the only factors associated with more fever of unknown origin were younger age and diagnosis in an earlier era.

**Table 3:** Predictors of the rates of microbiologically documented sterile site infection clinically documented infection and fever of unknown origin per course among intensive treatment courses (N = 101)

	**Microbiological sterile site**		**Clinically documented**		**Fever of unknown origin**	
	**Rate ratio**	***P *value**	**Rate ratio**	***P *value**	**Rate ratio**	***P *value**
	**(CI)**		**(CI)**		**(CI)**	
**Child characteristics at diagnosis**						
Age in years	0.92	0.082	0.99	0.748	0.92	0.025
	(0.84, 1.01)		(0.92, 1.06)		(0.85, 0.99)	
Obese vs. non-obese^1^	0.92	0.891	0.60	0.268	0.40	0.103
	(0.29, 2.93)		(0.24, 1.48)		(0.13, 1.20)	
**Treatment characteristics**						
APL-specific treatment protocol	0.77	0.643	2.63	0.127	0.69	0.496
	(0.25, 2.37)		(0.76, 9.10)		(0.24, 2.01)	
Registered on study	1.91	0.162	1.24	0.553	0.49	0.385
	(0.77, 4.70)		(0.62, 2.48)		(0.10, 2.43)	
Diagnosed prior to January 1, 2000	3.18	0.030	1.22	0.691	2.87	0.018
	(1.12, 9.04)		(0.46, 3.18)		(1.20, 6.86)	
Cumulative dose of cytarabine (g/m^2^)	1.09	0.008	0.97	0.522	0.99	0.763
	(1.02, 1.16)		(0.88, 1.07)		(0.90, 1.08)	
**Course characteristics**						
Neutropenia (ANC <0.5 ×10^9^) at start of course	0.99	0.990	5.68	<0.0001	1.36	0.436
	(0.32, 3.06)		(2.42, 13.31)		(0.63, 2.93)	
Greater than 15 days with neutropenia	2.04	0.208	4.18	0.001	2.01	0.138
	(0.67, 6.21)		(1.81, 9.62)		(0.80, 5.07)	
Days receiving corticosteroids	1.04	0.064	1.03	0.075	1.01	0.555
	(1.00, 1.09)		(1.00, 1.07)		(0.97, 1.05)	

Abbreviations: *APL* acute promyelocytic leukemia, *ANC* absolute neutrophil count, *CI* confidence interval; ^1^ Obesity only available for children ≥ 2 years of age.

When evaluating course characteristics, diagnosis prior to January 1, 2000 was correlated with cumulative cytarabine dose (Spearman r = 0.385, P < 0.0001) and thus, multiple regression was not conducted for microbiologically documented sterile site infection. Similarly, neutropenia at the start of the course was correlated with prolonged duration of neutropenia (Spearman r = 0.469, P < 0.0001) and thus, multiple regression was also not conducted for clinically documented infection. Age (RR 0.93, 95% CI 0.87 to 0.99; P = 0.033) and diagnosis in an earlier era (RR 2.53, 95% CI 1.17 to 5.46; P = 0.019) were both independently associated with fever of unknown origin.

Of the specific infections documented, Gram-positive bacterial infections were more common than Gram-negative or fungal infections (Table 4). Among the 3 fungal infections, 2 were from a sterile site (both *Candida albicans*) while 1 was from a non-sterile site (*Alternaria* species). Only the patient with *Alternaria* infection received antifungal prophylaxis with fluconazole.

**Table 4:** Microbiologically documented infections observed during therapy (N = 29)

**Organism**	**n (%)**
Sterile site bacteria*	
Gram positive	13 (44.8)
Viridans group streptococci	6 (20.7)
Coagulase negative staphylococci	5 (17.2)
*Enterococcus faecalis*	1 (3.4)
*Mycoplasma pneumoniae*	1 (3.4)
Gram Negative	4 (13.8)
*Escherichia coli*	2 (7.0)
*Klebsiella pneumoniae*	1 (3.4)
*Enterobacter cloacae*	1 (3.4)
Fungus*	3 (10.3)
*Candida albicans*	2 (7.0)
*Alternaria* NOS	1 (3.4)
Virus*	9 (31.0)
Herpes simplex virus	2 (7.0)
Respiratory syncytial virus	1 (3.4)
Torovirus	4 (13.8)
Other^1^	2 (7.0)

Abbreviation: *NOS* not otherwise specified;

* For bacterial infections, only sterile site positive cultures are shown. For fungi and viruses, both sterile and non-sterile site positive cultures are shown;

^1^ Others were adenovirus (n = 1) and influenza A (n = 1).

## Discussion

We found that among children with APL, one third experienced at least one sterile site bacterial infection throughout treatment and 14% of intensive chemotherapy courses were associated with a microbiologically documented sterile site infection. Invasive fungal infection was rare but did occur. Further, we found that the risk of infection has decreased over time.

These infection rates appear lower than those described for pediatric non-APL AML treatment protocols. In an evaluation of children enrolled on the Children’s Cancer Group 2961 protocol, more than 60% of children experienced a microbiologically documented infection during each course of therapy [15]. In an analysis of AAML0531, the most recently completed Children’s Oncology Group phase 3 AML trial, over 80% of children experienced at least one sterile site bacterial infection while 14% experienced at least one sterile site fungal infection throughout chemotherapy. The risk of sterile site bacterial infection was 30 to 60% per course [16].

Within our Canadian study focused on infections in AML, the cumulative risk of bacteremia was 54.3% for children with non-APL AML [7], compared with a 36.4% for any sterile site bacterial infection in this APL-specific study [7]. When comparing the risk by course, the risk of sterile site microbiologically documented infection was 24.5% in non-APL AML in comparison to 13.9% among intensively treated courses in APL [7]. When put together, these data suggest that the overall risk of invasive infections among children with APL may be less than that experienced by children with non-APL AML. Nonetheless, many children experienced invasive infections and there were two episodes of candidemia and one infection-related death. Furthermore, we did not find that APL-specific treatment protocol was associated with significantly fewer sterile site microbiologically documented infection in regression analysis although power was limited. Consequently, children with APL may benefit from aggressive supportive care similar to children with non-APL AML, at least during intensive courses of chemotherapy.

The rates of infection and infectious deaths have been variable on adult-predominated APL clinical trials [17-19]. However, almost none of these studies focused on infection outcomes. Girmenia et al [20]. evaluated 89 adult and pediatric patients with APL treated with the AIDA (all-trans retinoic acid plus idarubicin) protocol. Microbiologically documented infection occurred in 37.4% of patients. Fungal infections were rare. They compared bloodstream infections in APL patients with other AML patients and concluded that the incidence of total septicemia, fungemia and coagulase-negative staphylococci were significantly lower in APL. One infection-related death was observed. Consequently, our results are similar to this study.

In our study of pediatric non-APL AML patients, we found that exposure to corticosteroids was the most important factor associated with infection outcomes [7]. In contrast, within this analysis, we found that the association between duration of corticosteroids and rate of microbiologically documented infection was not statistically significant (rate ratio 1.04, 95% confidence interval 1.00 to 1.09; P = 0.064). However, given the observed lower confidence interval of 1.00, it is possible that this analysis was underpowered to demonstrate an association.

In this study, 6% of courses were complicated by sepsis. It is difficult to know how many courses were, in fact, truly complicated by infection-related sepsis since sepsis and differentiation syndrome associated with all-trans-retinoic acid have many similar features. Jeddi and colleagues recently highlighted how differentiation syndrome, which is thought to be mediated through inflammatory cytokines generated by APL cells, may not be distinguishable from sepsis [21]. Nonetheless, our report does provide reassuring data since we have likely over-estimated the rate of sepsis and the true rate may be lower.

The strength of our report is that we conducted a population-based analysis of a very rare sub-type of AML in children and we were able to measure infections very accurately because of the use of consistent and specifically trained personnel. The inclusion of very ill patients at presentation is another important strength of this study. However, our report must be interpreted in light of its limitations. APL treatments were heterogeneous. Second, supportive care strategies were also variable between centers. Finally, the sample size was small and many analyses were performed; consequently the results should be considered hypothesis generating rather than definitive.

## Conclusions

In summary, one third of children with APL experienced at least one sterile site bacterial infection throughout treatment and 14% of intensive chemotherapy courses were associated with a microbiologically documented sterile site infection. Infection rates in pediatric APL may be lower compared to non-APL AML although these children may still benefit from aggressive supportive care during intensive chemotherapy. Aggressive support care could include mandatory hospitalization during neutropenia and prophylactic antibacterial and antifungal strategies.

## Competing interest

There are no conflicts of interest to declare.

## Authors’ contributions

All authors contributed to data collection and manuscript writing. JB and LS contributed to data analysis and interpretation, and BG, MCE and LS also contributed to study conception and design. All authors have approved the final version of the manuscript.

## Pre-publication history

The pre-publication history for this paper can be accessed here:

http://www.biomedcentral.com/1471-2407/13/276/prepub

## Canadian infections in AML research group

David Dix (PI), Buffy Menjou (CRA) and Nita Takeuchi (CRA) from British Columbia Children’s Hospital; Kent Stobart (PI), Brenda Ennis (CRA) and Linda Churcher (CRA) from Stollery Children’s Hospital; Victor Lewis (PI), Janice Hamilton (CRA) and Karen Mazil (CRA) from Alberta Children’s Hospital; Sonia Cellot (PI), Dominique Lafreniere (CRA) and Catherine Desjean (CRA) from Hospital Sainte-Justine; Victoria Price (PI), Tina Bocking (CRA), Lynn Russell (CRA) and Emily Murray (CRA) from IWK Health Centre; Lynette Bowes (PI) and Gale Roberts (CRA) from Janeway Child Health Centre; Carol Portwine (PI) and Sabrina Siciliano (CRA) from McMaster Children’s Hospital at Hamilton Health Sciences; Joseph Beyene (Collaborator) from McMaster University; Mariana Silva (PI) from the Cancer Centre of Southeastern Ontario at Kingston; Rochelle Yanofsky (PI), Rebekah Hiebert (CRA) and Krista Mueller (CRA) from CancerCare Manitoba; Shayna Zelcer (PI), Martha Rolland (CRA) and Julie Nichols (CRA) from London Health Sciences; Donna Johnston (PI) and Elaine Dollard (CRA) from Children’s Hospital of Eastern Ontario; David Mitchell (PI), Martine Nagy (CRA) and Margaret Hin Chan (CRA) from Montreal Children’s Hospital; Bruno Michon (PI), Josee Legris (CRA) and Marie-Christine Gagnon (CRA) from Hospitalier Universitaire de Quebec; Josee Brossard (PI) and Lise Bilodeau (CRA) from Centre Hospitalier Universitaire de Sherbrooke; Lillian Sung (PI), Biljana Gillmeister (CRA), Marie-Chantal Ethier (CRA), Renee Freeman (Collaborator), Jeffrey Traubici (Collaborator), and Upton Allen (Collaborator) from The Hospital for Sick Children.
